# Clinicopathological characteristics and prognosis of pulmonary large cell neuroendocrine carcinoma aged ≥65 years

**DOI:** 10.7717/peerj.6824

**Published:** 2019-05-20

**Authors:** Ling Cao, Ling Zhao, Min Wang, Xu he Zhang, Zhu-chun Yang, Yun-peng Liu

**Affiliations:** 1 Department of Radiation Oncology, Cancer Hospital of Jilin Province, Changchun, Jilin, China; 2 Department of Pathology, Cancer Hospital of Jilin Province, Changchun, Jilin, China; 3 Department of Head and Neck Surgery, Cancer Hospital of Jilin Province, Changchun, China; 4 Department of Thoracic Surgery, First Hospital of Jilin University, Changchun, Jilin, China

**Keywords:** Pulmonary large cell neuroendocrine carcinoma, Aged, Prognosis, SEER

## Abstract

**Objective:**

The present study was designed to better characterize the clinicopathological features and prognosis in patients aged ≥65 years with pulmonary large cell neuroendocrine carcinoma (LCNEC).

**Methods:**

Eligible patients with pulmonary LCNEC were retrieved from the Surveillance, Epidemiology, and End Results database between January 2004 and December 2013. The primary endpoints included cancer-specific survival (CSS) and overall survival (OS).

**Results:**

Data of 1,619 eligible patients with pulmonary LCNEC were collected. These patients were subsequently categorized into two groups: 890 patients in the older group (age ≥65 years), and 729 in the younger group (age <65 years). More patients were of white ethnicity, stage I, married, and with tumor size <5 cm in the older group in comparison to the younger group. However, there were a significantly lower proportion of patients undergoing surgery, chemotherapy, and radiotherapy in the older group. The 5-year CSS rates of the younger group and older group were 23.94% and 17.94% (*P* = 0.00031), respectively, and the 5-year OS rates were 20.51% and 13.47% (*P* < 0.0001), respectively. Multivariate analyses indicated that older age (CSS: HR 1.20, 95% CI [1.07–1.36], *P* = 0.0024; OS: HR 1.26, 95% CI [1.12–1.41], *P* < 0.0001) was an independent risk factor for poor prognosis. The mortality risk of the elderly increased in almost every subgroup, especially in OS. Finally, significant predictors for better OS and CSS in patients over age 65 included tumor size <5 cm, lower stage, and receiving surgery, chemotherapy, or radiotherapy.

**Conclusion:**

The prognosis of patients aged ≥65 years with pulmonary LCNEC was worse than that of younger patients. However, active and effective therapy could significantly improve the survival of older patients with pulmonary LCNEC.

## Introduction

Large cell neuroendocrine carcinoma (LCNEC) has been considered a rare pulmonary malignancy, accounting for only 2–3% of all diagnosed lung cancers (Fasano et al., 2015). LCNEC was initially categorized into the spectrum of pulmonary neuroendocrine tumors in 1991. Prior to this, it was classified as a high-grade atypical carcinoid tumor (Travis et al., 1991). Afterward, during 1999 and 2004, the World Health Organization (WHO) admitted that LCNEC was a variant of large cell carcinoma, belonging to neuroendocrine tumors and one of the non-small cell lung cancer (NSCLC). Furthermore, pulmonary LCNEC was classified as a neuroendocrine carcinoma in combination with small cell lung cancer (SCLC), typical carcinoid, and atypical carcinoid according to the WHO fourth edition Classification of Lung Tumors (Wood et al., 2018).

Increasing life expectancy within the generalized population has resulted in a rising incidence of elderly patients with lung cancer. Additionally, roughly 47% of patients with lung cancer in the US are over 70 years of age, and 14% were over 80 years (Owonikoko et al., 2007). In comparison with younger patients, older populations diagnosed with NSCLC are commonly labeled unfit for treatment due to the increasing treatment-related toxicity. Furthermore, there were more consequential deterioration and comorbidities associated with worse life quality. Therefore, elderly patients are excluded from various studies, whose outcomes are, therefore, not suitable for the elderly (Hutchins et al., 1999; Lewis et al., 2003; Talarico, Chen & Pazdur, 2004).

Clinically, pulmonary LCNEC is thought to be an aggressive malignancy with higher risks of recurrence and metastasis in comparison with other types of NSCLC. Moreover, older patients with pulmonary LCNEC are often considered to harbor worse prognosis (Fasano et al., 2015). However, to the best of our knowledge, studies concerning the comparison between the elder group and the younger group have never been undertaken. In order to provide a better understanding of pulmonary LCNEC in the elderly for clinicians, the present study was performed and analyzed to investigate its clinicopathological characteristics, prognosis, and risk factors.

## Materials and Methods

### Ethics statement

The Surveillance, Epidemiology, and End Results (SEER) program has developed a comprehensive, population-based database that was established in 1973, and gets updated annually (Duggan et al., 2016). The database includes nearly 30% of US population across multiple geographic areas (Cronin, Ries & Edwards, 2014). The SEER Research Data Agreement was signed for accessing SEER information with the use of reference number 16462-Nov2016. We proceeded to perform research methods for obtaining information while following approved guidelines. Data analysis from this database is considered to be non-human subjects by the Office for Human Research Protection as part of the US Department of Health and Human Services, because patient data was anonymized and publicly available. For these reasons, no ethical approval was required.

### Study population

The SEER database was used to obtain patient data (submission, November 2016). On March 6, 2018 the SEER State v8.3.5 tool was released, which was utilized for selecting and choosing eligible patients for this study. In addition, our study focused on the period between January 2004 and December 2013. The inclusion criteria were as follows: over 18 years of age at diagnosis; LCNEC was pathologically confirmed using histology (ICD-O-38013/3); restriction site recoded in ICD-O-3 (International Classification of Diseases for Oncology, Third Edition)/WHO 2008 to “Lung and Bronchus.” Meanwhile, the exclusion criteria included: (1) under 18 years of age; (2) multiple primary cancer diagnosis; (3) without survival data; (4) without pathological confirmation based on histology; (5) because LCNEC is a high grade neuroendocrine lung tumors, we excluded patients with low grade pathology (Grade I and Grade II); (6) without surgical method; (7) patients that did not have sixth American Joint Committee on Cancer (AJCC) staging. The patients that met these criteria were included in the SEER primary cohort.

### Covariates

Demographic and clinical variables were extracted from the SEER database, including age at diagnosis, sex, race, marital status, primary site, laterality, grade, tumor size, T, N, and M stage, surgery, chemotherapy, radiotherapy, follow-up information. Tumor size was a continuous variable which was transformed into a categorical variable on the basis of recognized cut-off values. We used the sixth edition AJCC TNM staging system, and we limited our research to between 2004 and 2013, because it was published in 2004. The endpoints of this study were cancer-specific survival (CSS) and overall survival (OS). CSS was defined as the interval from diagnosis to the most recent follow-up date or date of death caused by pulmonary LCNEC. OS was defined as the interval from diagnosis to the most recent follow-up date, or date of death. Using SEER 2016, a predetermined cut-off date was decided, which contained information about deaths until 2014. Therefore, the study used a cut-off date of December 31, 2014.

### Statistical analysis

Baseline continuous and categorical variables are presented as a median with range and numbers with percentages, respectively. Meanwhile, clinicopathological characteristics were compared with Fisher’s exact tests or Pearson’s χ^2^ as deemed appropriate. Additionally, Kaplan–Meier method was utilized for estimating patient survival rate. Survival differences between the groups were evaluated using the log-rank tests. Univariate and multivariate COX proportional hazard regression models were utilized to evaluate risk of mortality and conduct subgroup analyses. Variables that were deemed to be of potential importance in univariate analysis (*P* < 0.05) or previously considered to be prognostic factors were included in multivariate analysis. All of our statistical analysis conducted used SPSS software (version 19.0; SPSS Inc., Chicago, IL, USA). Our statistical significance level was set to *P* < 0.05.

## Results

### Patient screening process

**Figure 1:**
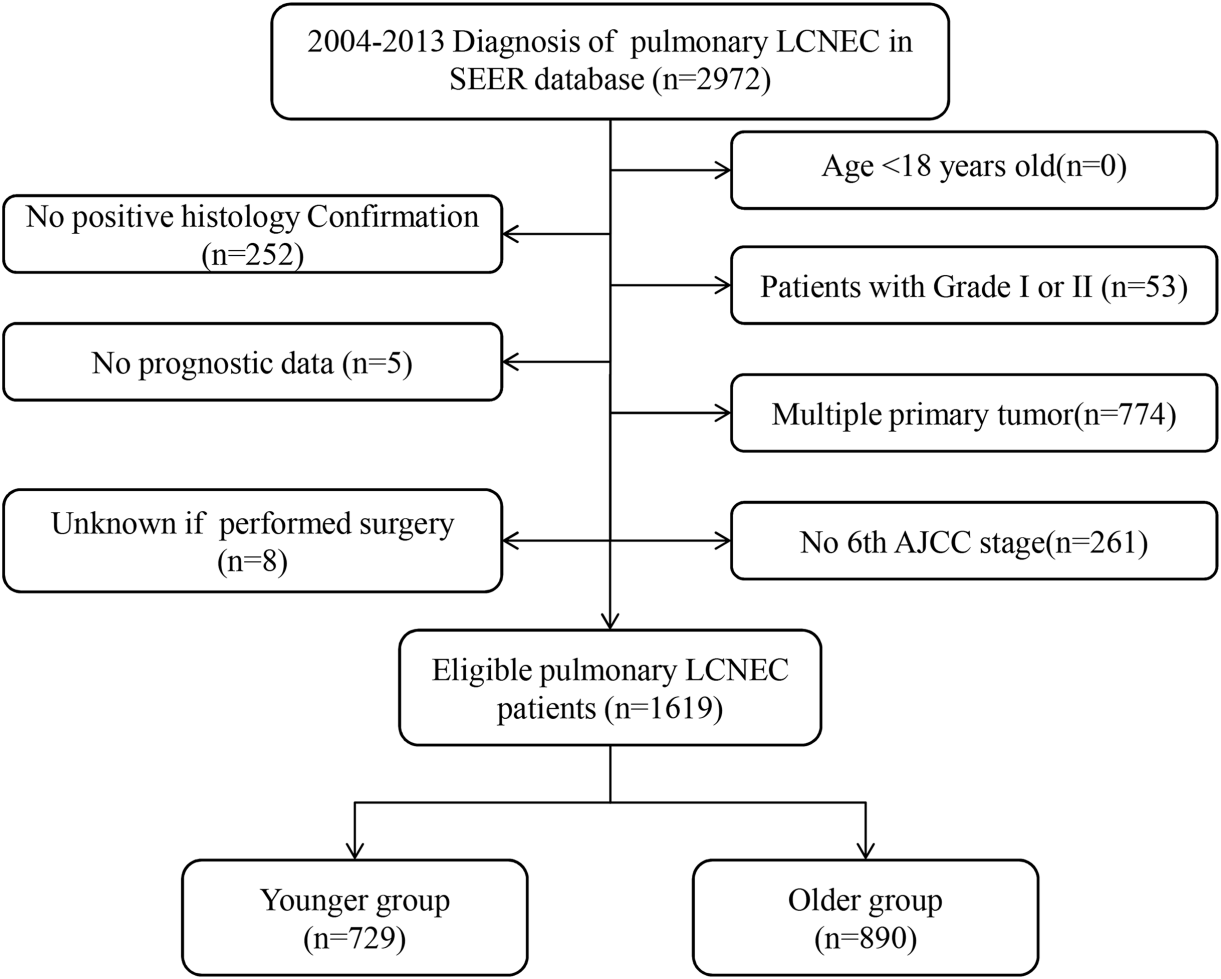
Flow chart for screening eligible patients.

A total of 1,619 eligible patients with pulmonary LCNEC were included in this study. The screening process was described in Fig. 1. Median age at diagnosis of all patients was 66 years (range, 18–94 years). Using median age and previous clinical studies as guides (Brueckl et al., 2018; Feliciano et al., 2018; Hutchins et al., 1999; Lembicz et al., 2018; Lewis et al., 2003), we divided the included patients into two groups based on age: younger (aged <65 years) (*N* = 729), and older (aged ≥65 years) (*N* = 890) patients.

### Clinicopathological characteristics

The clinicopathological features of all enrolled patients were summarized in Table 1. In the younger group, the median age was 57 years, while the median age of the older group (≥65 years) was 73 years. In the older group, more patients were white (*P* = 0.004), married (*P* < 0.001), of stage I (*P* = 0.022), with tumor size <5 cm (*P* = 0.013); while with significantly lower proportion of surgery (*P* < 0.001), chemotherapy (*P* < 0.001), and radiotherapy (*P* < 0.001).

**Table 1:** Clinicopathological characteristics of patients with pulmonary large cell neuroendocrine carcinoma according to age groups.

Variables	<65 years (*N* = 729)	≥65 years (*N* = 890)	*P*-Value
	*N* (%)	*N* (%)	
Median age (range)	57 (18–64)	73 (65–94)	
Sex			0.524
Female	321 (44.03%)	406 (45.62%)	
Male	408 (55.97%)	484 (54.38%)	
Race			0.004
White	591 (81.07%)	774 (86.97%)	
Black	108 (14.81%)	80 (8.99%)	
API/AI	29 (3.98%)	34 (3.82%)	
Unknown	1 (0.14%)	2 (0.22%)	
Marital status			<0.001
Married	409 (56.10%)	679 (76.29%)	
Unmarried	292 (40.05%)	186 (20.90%)	
Unknown	28 (3.84%)	25 (2.81%)	
Primary site			0.761
Main bronchus	25 (3.43%)	35 (3.93%)	
Peripheral	641 (87.93%)	772 (86.74%)	
Unknown	63 (8.64%)	83 (9.33%)	
Grade			0.325
Grade III	293 (40.19%)	331 (37.19%)	
Grade IV	81 (11.11%)	116 (13.03%)	
Unknown	355 (48.70%)	443 (49.78%)	
Laterality			0.221
Left	276 (37.86%)	373 (42.00%)	
Right	435 (59.67%)	497 (55.97%)	
Bilateral	18 (2.47%)	18 (2.03%)	
AJCC stage			0.022
I	179 (24.55%)	256 (28.76%)	
II	65 (8.92%)	49 (5.51%)	
III	145 (19.89%)	185 (20.79%)	
IV	340 (46.64%)	400 (44.94%)	
Tumor size			0.013
<5 cm	420 (57.61%)	566 (63.60%)	
≥5 cm	235 (32.24%)	228 (25.62%)	
Unknown	74 (10.15%)	96 (10.79%)	
Surgery style			<0.001
No surgery	416 (57.06%)	548 (61.57%)	
Segmentectomy/wedge resection	46 (6.31%)	98 (11.01%)	
Lobectomy/Bilobectomy	228 (31.28%)	215 (24.16%)	
Pneumonectomy	39 (5.35%)	29 (3.26%)	
Chemotheray			<0.001
No/unknown	291 (39.92%)	499 (56.07%)	
Yes	438 (60.08%)	391 (43.93%)	
Radiotherapy			<0.001
No/unknown	411 (56.38%)	594 (66.74%)	
Yes	318 (43.62%)	296 (33.26%)	

**Note:**

API/AI, American Indian/AK Native, Asian/Pacific Islander.

### The impact of age on the prognosis

Univariate analysis revealed that age at diagnosis, sex, primary site, laterality, tumor size, AJCC stage, surgery, chemotherapy, and radiotherapy were associated with survival in patients with pulmonary LCNEC (Table 2). Subsequent multivariate analysis indicated that older age (≥65 years) (CSS: HR 1.20, 95% CI [1.07–1.36], *P* = 0.0024; OS: HR 1.26, 95% CI [1.12–1.41], *P* < 0.0001) was an independent prognostic risk factor for CSS and OS (Table 3).

**Table 2:** Univariate analyses for prognostic factors in pulmonary large cell neuroendocrine carcinoma.

Variables	Statistics	CSS		OS	
	*N* (%)	HR [95% CI]	*P*-Value	HR [95% CI]	*P*-Value
Age					
<65	729 (45.03%)	1.0		1.0	
≥65	890 (54.97%)	1.23 [1.10–1.38]	0.0004	1.30 [1.17–1.46]	<0.0001
Sex					
Female	727 (44.90%)	1.0		1.0	
Male	892 (55.10%)	1.18 [1.05–1.32]	0.0060	1.20 [1.07–1.33]	0.0014
Race					
White	1,365 (84.31%)	1.0		1.0	
Black	188 (11.61%)	0.95 [0.79–1.13]	0.5558	0.96 [0.81–1.13]	0.5992
API/AI	63 (3.89%)	1.01 [0.76–1.35]	0.9371	1.00 [0.76–1.31]	0.9872
Unknown	3 (0.19%)	0.46 [0.06–3.27]	0.4373	0.41 [0.06–2.93]	0.3757
Marital status					
Married	1,088 (67.20%)	1.0		1.0	
Unmarried	478 (29.52%)	1.08 [0.95–1.23]	0.2351	1.06 [0.94–1.19]	0.3630
Unknown	53 (3.27%)	0.97 [0.69–1.36]	0.8513	1.04 [0.76–1.43]	0.7921
Primary site					
Main bronchus	60 (3.71%)	1.0		1.0	
Peripheral	1,413 (87.28%)	0.45 [0.34–0.59]	<0.0001	0.45 [0.34–0.58]	<0.0001
Unknown	146 (9.02%)	0.95 [0.69–1.32]	0.7680	0.93 [0.69–1.27]	0.6619
Grade					
Grade III	624 (38.54%)	1.0		1.0	
Grade IV	197 (12.17%)	1.09 [0.89–1.32]	0.4158	1.10 [0.91–1.32]	0.3318
Unknown	798 (49.29%)	1.76 [1.55–1.99]	<0.0001	1.70 [1.51–1.91]	<0.0001
Laterality					
Left	649 (40.14%)	1.0		1.0	
Right	932 (57.64%)	1.10 [0.98–1.24]	0.1209	1.10 [0.98–1.23]	0.1015
Bilateral	36 (2.23%)	1.58 [1.08–2.30]	0.0174	1.55 [1.08–2.21]	0.0164
AJCC stage					
I	435 (26.87%)	1.0		1.0	
II	114 (7.04%)	2.17 [1.64–2.87]	<0.0001	1.87 [1.45–2.42]	<0.0001
III	330 (20.38%)	3.00 [2.46–3.66]	<0.0001	2.55 [2.14–3.04]	<0.0001
IV	740 (45.71%)	7.14 [5.99–8.51]	<0.0001	5.63 [4.82–6.59]	<0.0001
Tumor size					
<5 cm	986 (60.90%)	1.0		1.0	
≥5 cm	463 (28.60%)	1.92 [1.68–2.18]	<0.0001	1.80 [1.59–2.03]	<0.0001
Unknown	170 (10.50%)	3.14 [2.63–3.76]	<0.0001	2.88 [2.42–3.43]	<0.0001
Surgery style					
No surgery	964 (59.54%)	1.0		1.0	
Segmentectomy/wedge resection	144 (8.89%)	0.33 [0.26–0.41]	<0.0001	0.37 [0.30–0.46]	<0.0001
Lobectomy/Bilobectomy	443 (27.36%)	0.20 [0.17–0.23]	<0.0001	0.23 [0.20–0.27]	<0.0001
Pneumonectomy	68 (4.20%)	0.34 [0.25–0.47]	<0.0001	0.40 [0.30–0.53]	<0.0001
Chemotheray					
No/unknown	790 (48.80%)	1.0		1.0	
Yes	829 (51.20%)	1.02 [0.91–1.14]	0.7596	0.93 [0.83–1.03]	0.1759
Radiotherapy					
No/unknown	1,005 (62.08%)	1.0		1.0	
Yes	614 (37.92%)	1.39 [1.24–1.56]	<0.0001	1.28 [1.15–1.43]	<0.0001

**Note:**

API/AI, American Indian/AK Native, Asian/Pacific Islander.

**Table 3:** Multivariate analyses for different age group in pulmonary large cell neuroendocrine carcinoma.

Variables	Non-adjusted		Adjust*	
	HR [95% CI]	*P*-Value	HR [95% CI]	*P*-Value
CSS				
<65 years	1.0		1.0	
≥65 years	1.23 [1.10–1.38]	0.0004	1.20 [1.07–1.36]	0.0024
OS				
<65 years	1.0		1.0	
≥65 years	1.30 [1.17–1.46]	<0.0001	1.26 [1.12–1.41]	<0.0001

**Note:**

* Adjust model adjust for: sex, primary site, laterality, AJCC stage, tumor size, surgery style, chemotherapy, radiotherapy.

### Survival analysis and subgroup analysis

**Figure 2:**
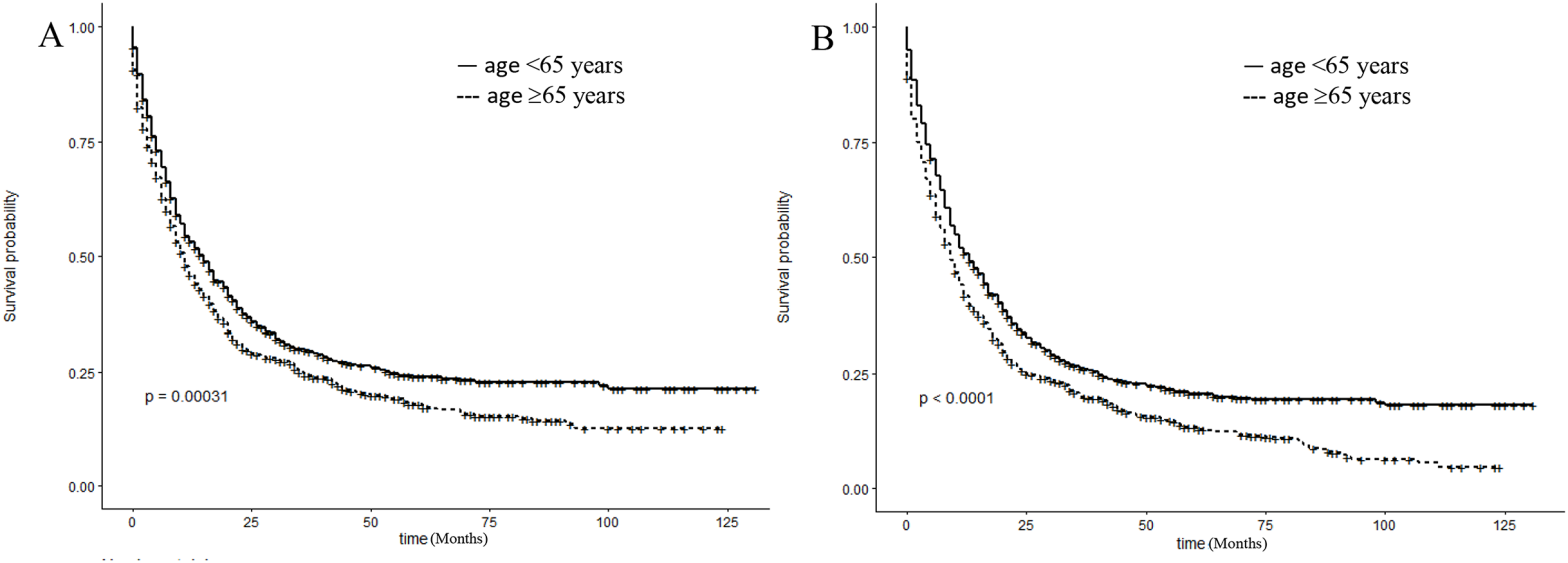
Kaplan–Meier survival plots for different age group patients showing (A) cancer-specific survival (CSS) and (B) overall survival (OS) (log-rank tests).

**Figure 3:**
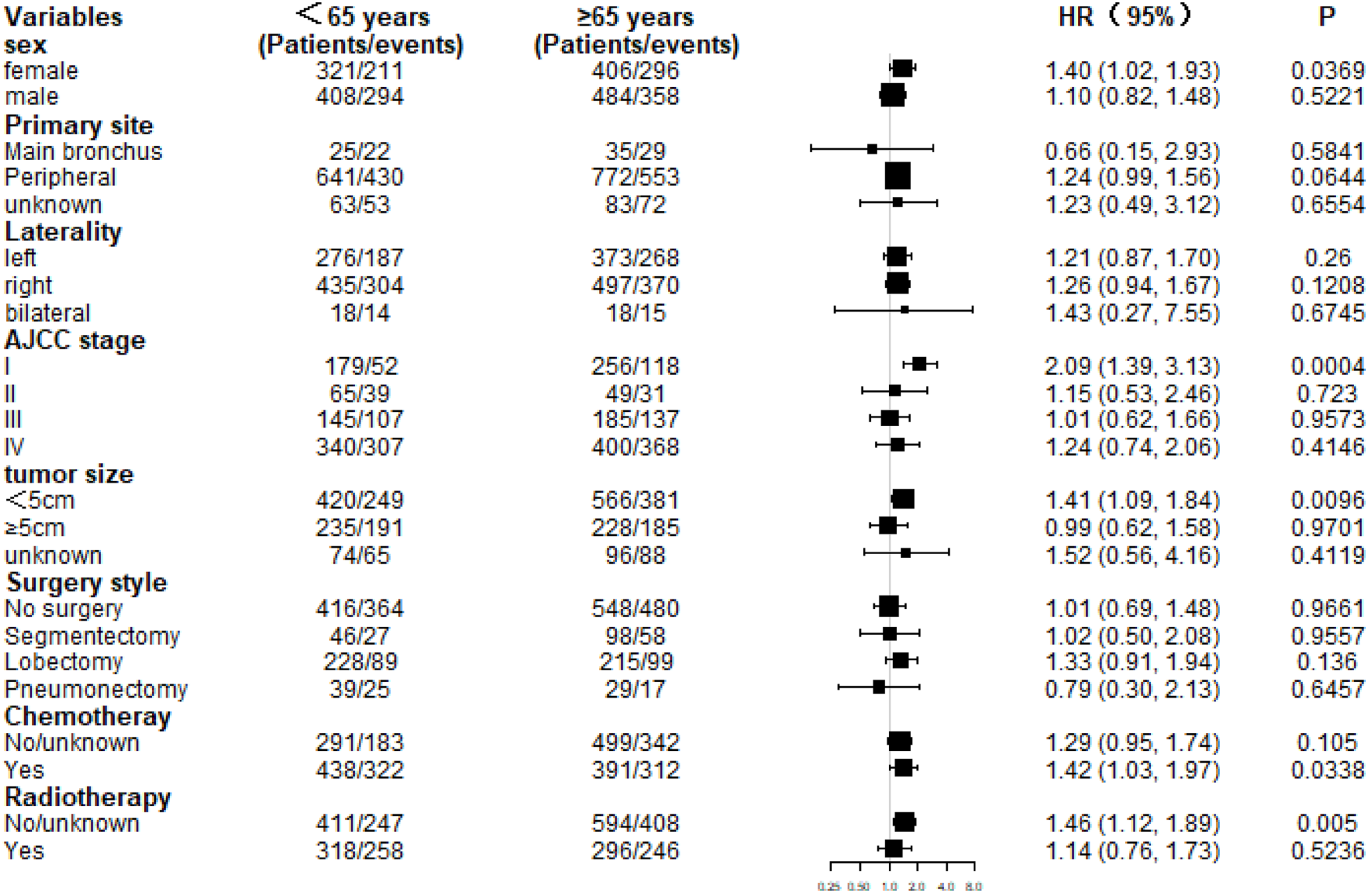
Subgroup analysis of cancer-specific survival (CSS) between the two age groups.

**Figure 4:**
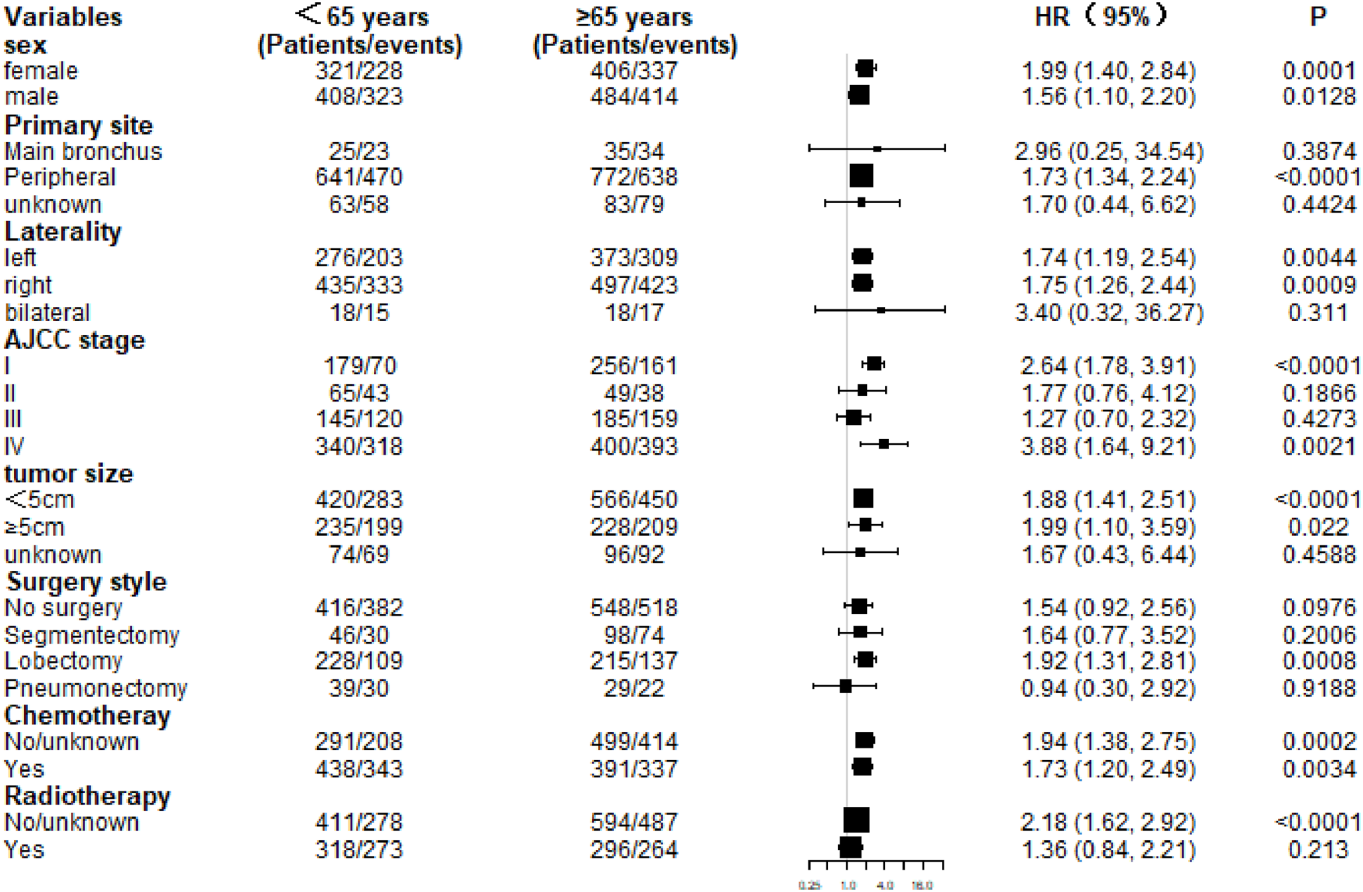
Subgroup analysis of overall survival (OS) between the two age groups.

The 5-year CSS rates of the younger group and older group were 23.94% and 17.94% (*P* = 0.00031), respectively, and the 5-year OS rates were 20.51% and 13.47% (*P* < 0.0001), respectively (Fig. 2). Subgroup analysis revealed that CSS in the older group was lower than that in the younger group, consistent with the findings in the overall study population. Additionally, CSS was statistically significant in the subgroups stratified by female, AJCC stage I, tumor size <5 cm, and chemotherapy, without radiotherapy (Fig. 3). Meanwhile, patients of the older group in nearly all subgroups harbored significantly lower OS, except those with primary tumor location in main bronchus, bilateral tumor, AJCC stage II, stage III, and undergoing segmentectomy, pneumonectomy, and radiotherapy (Fig. 4).

### Prognostic survival factors of older group patients

Univariate and multivariate analyses found that tumor size, AJCC stage, surgery, chemotherapy, radiotherapy were independent risk factors for prognosis in the older group (≥65 years) (Table 4). Moreover, patients with tumor ≥5 cm and advanced stage had worse prognosis. Additionally, surgery, chemotherapy, and radiation significantly prolonged the survival duration of older patients.

**Table 4:** Univariate and multivariate analyses of cancer special survival (CSS) and overall survival (OS) in the older group.

Variables	CSS			OS		
	Univariate analysis	Multivariate analysis		Univariate analysis	Multivariate analysis	
	*P*-Value	HR [95% CI]	*P*-Value	*P*-Value	HR [95% CI]	*P*-Value
Sex			NI			NI
Female						
Male	0.7211			0.3004		
Race			NI			NI
White						
Black	0.5636			0.9295		
API/AI	0.9719			0.4144		
Unknown	0.4769			0.2290		
Marital status			NI			NI
Married						
Unmarried	0.9927			0.2889		
Laterality			NI			NI
Left						
Right	0.3915			0.3650		
Bilateral	0.2956			0.2253		
Primary site			NI			NI
Main bronchus						
Peripheral	0.1541			0.0537		
Grade			NI			NI
Grade III						
Grade IV	0.8141			0.7339		
Tumor size						
<5 cm		Reference			Reference	
≥5 cm	0.0001	1.56 [1.29–1.88]	<0.0001	<0.0001	1.61 [1.35–1.93]	<0.0001
AJCC stage						
I		Reference			Reference	
II	0.0296	2.00 [1.33–3.00]	0.0008	0.0517	1.88 [1.31–2.70]	0.0007
III	<0.0001	2.36 [1.76–3.16]	<0.0001	<0.0001	2.21 [1.70–2.88]	<0.0001
IV	<0.0001	5.00 [3.75–6.66]	<0.0001	<0.0001	4.42 [3.41–5.74]	<0.0001
Surgery						
No surgery		Reference			Reference	
Segmentectomy/wedge resection	<0.0001	0.45 [0.33–0.63]	<0.0001	<0.0001	0.50 [0.37–0.67]	<0.0001
Lobectomy/Bilobectomy	<0.0001	0.33 [0.25–0.45]	<0.0001	<0.0001	0.38 [0.29–0.50]	<0.0001
Pneumonectomy	0.0008	0.37 [0.22–0.62]	0.0002	<0.0001	0.40 [0.25–0.63]	0.0003
Chemotheray						
No/unknown		Reference	<0.0001		Reference	
Yes	0.0002	0.39 [0.32–0.46]		0.1893	0.38 [0.32–0.45]	<0.0001
Radiation						
No/unknown		Reference			Reference	
Yes	<0.0001	0.75 [0.63–0.89]	0.0010	0.0058	0.71 [0.60–0.84]	<0.0001

**Note:**

HR, hazard ratio; 95% CI, 95% confidence index; API/AI, American Indian/AK Native, Asian/Pacific Islander; NI, not included in the multivariate survival analysis.

## Discussion

The incidence of pulmonary LCNEC is rare, representing only 3% of all types of diagnosed lung cancer. Thus, the published studies of pulmonary LCNEC commonly included limited patients (Brueckl et al., 2018; Carretta et al., 2000; Mazieres et al., 2002; Zacharias et al., 2003). In this study, we found that more LCNEC patients were white, married, of stage I, with tumor size <5 cm in the older group; and the proportion of patients undergoing surgery, radiotherapy, and chemotherapy were significantly lower. In addition, older age (≥65 years) was an independent prognostic risk of survival. Moreover, tumor size, AJCC stage, surgery, chemotherapy, radiotherapy were independent prognostic risk factors for older patients.

Pulmonary LCNEC is biologically aggressive, with poor prognosis (Fasano et al., 2015). The 5-year OS rate for LCNEC after resection has been reported to range from 13% to 57% (Liang et al., 2015; Varlotto et al., 2011; Younossian, Brundler & Totsch, 2002). Similarly, in our study, we found that the 5-year OS rate in the older patient group and the younger group as 13.47% and 20.51%, respectively, which are consistent with previous studies. Meanwhile, further subgroup analysis revealed that survival risk increased in almost all subgroups, especially in the OS.

There are limited studies assessing how age impacts prognosis due to the low incidence of pulmonary LCNEC. Kujtan et al. (2018) reported that patients over 70 had worse survival outcomes. Additionally, Wu et al. (2014) confirmed that age was a prognostic factor for pulmonary neuroendocrine tumors, which, however, only included 23 patients (5.7%) with pulmonary LCNEC. Herein, our study analyzed data from 1,619 patients diagnosed with pulmonary LCNEC, and found that those elderly patients harbored significantly worse survival outcomes.

Up to date, the standard therapeutic regimen for pulmonary LCNEC is still uncertain, especially for elderly patients. Nevertheless, it is universally accepted that primary surgery is still the first option in operable patients (Naidoo et al., 2016), which constitutes the principal way to obtain an accurate diagnosis (Fasano et al., 2015). In our research, surgery was an independent prognostic factor. The role of chemotherapy or radiotherapy in the treatment of pulmonary LCNEC also remains unclarified (Hiroshima & Mino-Kenudson, 2017). Dresler et al. (1997) reported no survival benefits from postoperative chemotherapy, radiation therapy, or both in patients with resected LCNEC. Shimada et al. (2012) demonstrated that overall response rate to the initial chemotherapy or chemo-radiotherapy and the survival outcomes of high-grade neuroendocrine carcinoma (HGNEC)—probable LCNEC were comparable to those of SCLC. In our study, we found that chemotherapy and radiotherapy were protective factors for elderly pulmonary LCNEC. However, we are still unaware of how to choose chemotherapy and chemotherapeutic regimen. Unfortunately, further analysis is not possible at present due to the inaccessible specific content of chemotherapy and radiotherapy. Nevertheless, it is necessary to cautiously choose the therapeutic regimen for elderly pulmonary LCNEC.

To our knowledge, it is the largest retrospective analysis on the prognostic effect of age in pulmonary LCNEC. Based on a large population, there were certain limitations that should be noted in our study. Firstly, as a retrospective study, we selected patients according to inclusion and exclusion criteria, which might result in the potential risk of selection bias. Secondly, there were some clinicopathological parameters associated with prognosis which were unavailable in the SEER database, such as surgical margin status or the specific dosage of chemotherapy and radiotherapy. Although retrospective studies may not have all the all putative parameters available, often, as in this case, our results are certainly of great clinical value. Bridging these research gaps will be a major focus in future research.

## Conclusion

In conclusion, our study found that the prognosis in patients aged ≥65 years with pulmonary LCNEC was worse than that of younger ones. However, active and effective therapy can significantly improve survival rates for elderly, and multidisciplinary treatment could provide more survival benefits for elderly patients. Our findings could provide a better understanding for clinicians of clinicopathological features and prognosis in patients over 65 years of age with pulmonary LCNEC for clinicians.

## Supplemental Information

10.7717/peerj.6824/supp-1 Supplemental Information 1 Supplemental Information.

## Additional Information and Declarations

### Data Availability

The following information was supplied regarding data availability: The raw data is available as a Supplemental File.
